# Supplementary material for: Targeted Elimination of Immunodominant B Cells Drives the Germinal Center Reaction toward Subdominant Epitopes
Source: Cell Rep. Author manuscript; Available in PMC 2018 Jan 17. (PMC5771444; doi:10.1016/j.celrep.2017.12.014)
Supplement: 1 [file NIHMS931302-supplement-1.pdf]

Cell Reports, Volume *21*

Supplemental Information

Targeted Elimination of Immunodominant B Cells Drives the Germinal Center Reaction toward Subdominant Epitopes

Murillo Silva, Thao H. Nguyen, Phaethon Philbrook, Matthew Chu, Olivia Sears, Stephen Hatfield, Robert K. Abbott, Garnett Kelsoe, and Michail V. Sitkovsky

Supplemental Figures:

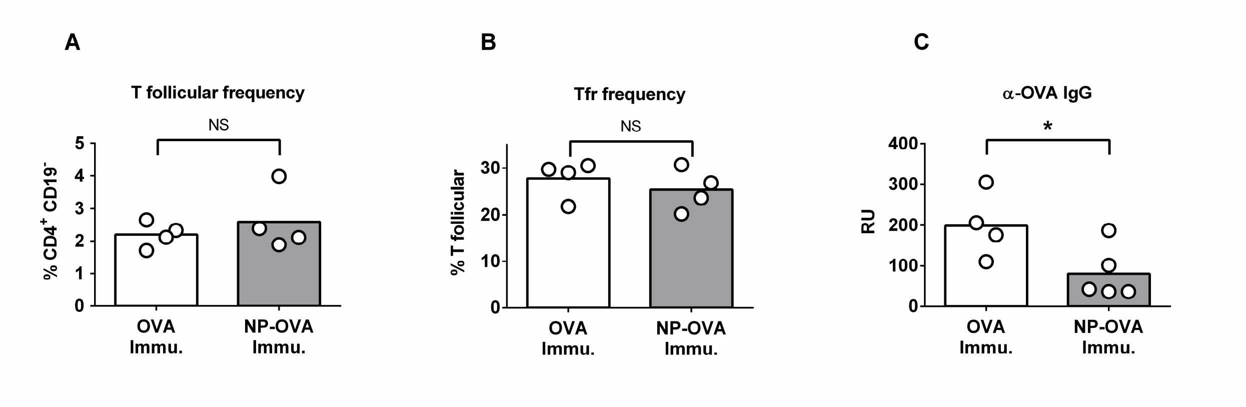

Figure S1. NP-OVA and OVA immunized mice have similar T follicular responses, related to Figure 1.
(A-B) Quantification of T follicular cells (A) and T follicular regulatory cells (B) $CD4^+$, $CD19^-$, $CXCR5^{hi}$, $PD1^{hi}$ and $CD4^+$, $CD19^-$, $CXCR5^{hi}$, $PD1^{hi}$ $Foxp3^+$.
(C) Anti-OVA serum IgG Ab response quantified by ELISA on day 10 after immunization.
Bars represent mean; NS, not significant; *p < 0.05 unpaired student's *t* test.

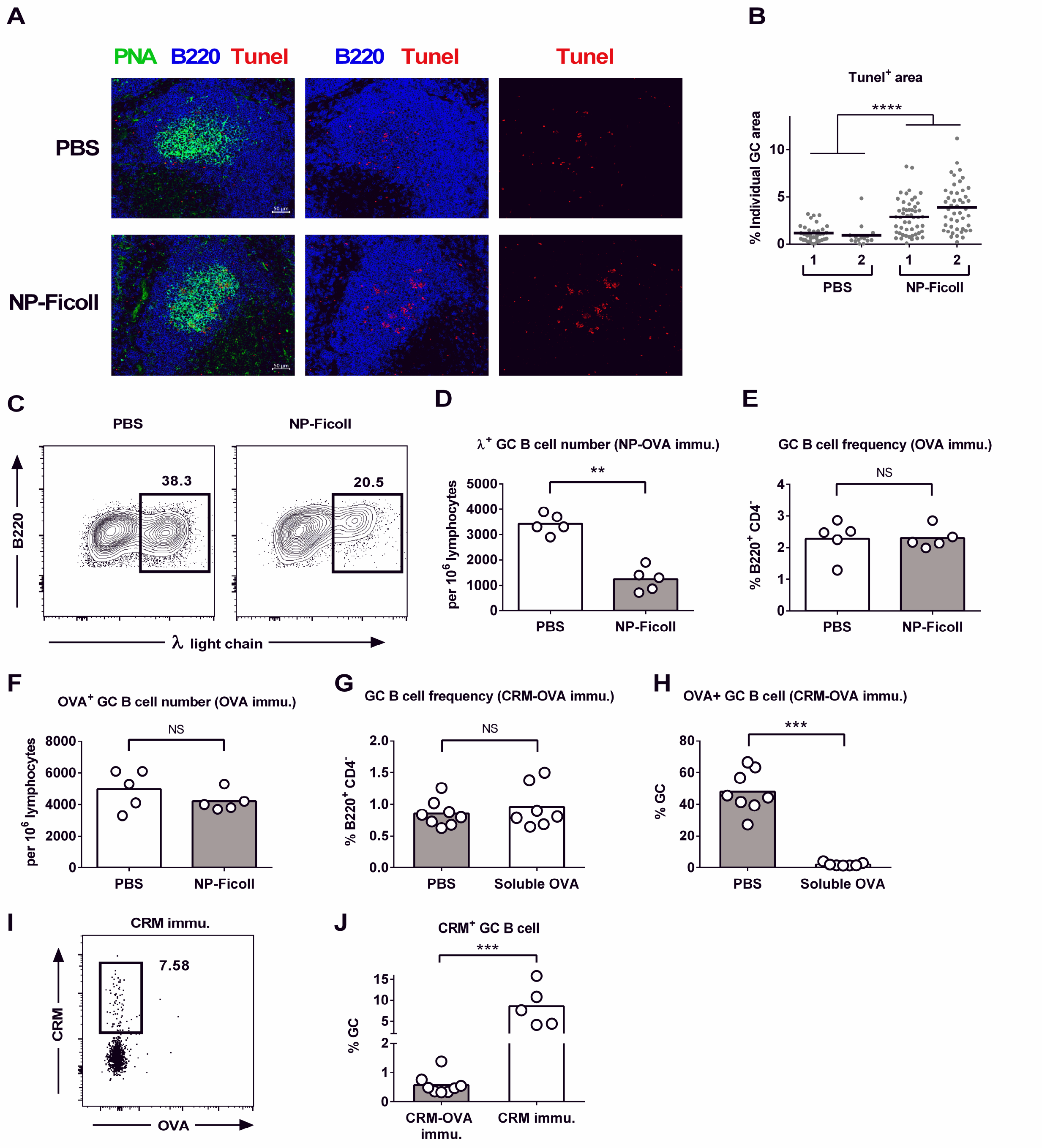

Figure S2. Antigen specific reduction of GC B cells, related to Figure 2.
(A-D) Mice were immunized and treated as in Figure 2A. (A) Representative tunel staining of GCs 6hr after NP Ficoll treatment (B) Quantification of tunel+ area in individual GCs of control on NP-Ficoll treated mice. (C) Representative gates of λ^+ GC B cells ($B220^+$, $GL7^+$, $CD4^-$, $CD38^{lo}$, λ^+) and quantification (D).
(E-F) Mice were immunized with unconjugated OVA in alum and treated with NP-Ficoll as in Figure 2A. Quantification of total GC B cells (E) and OVA-specific GC B cell number (F) after NP-Ficoll treatment (day 9).
(G, H) Mice were immunized i.p. with 1µg of CRM-OVA then treated with soluble OVA or PBS on day7. Spleens were dissected 12 days after immunization. (G) Quantification of total GC frequency and OVA specificity (H)
(I, J) Representative flow plot of mice immunized with unconjugated CRM and quantification of GC specificity (day 12) (J). Bars represent mean; NS, not significant; $**p < 0.01$, $***p < 0.001$, $****p < 0.0001$ unpaired Welch's *t* test.

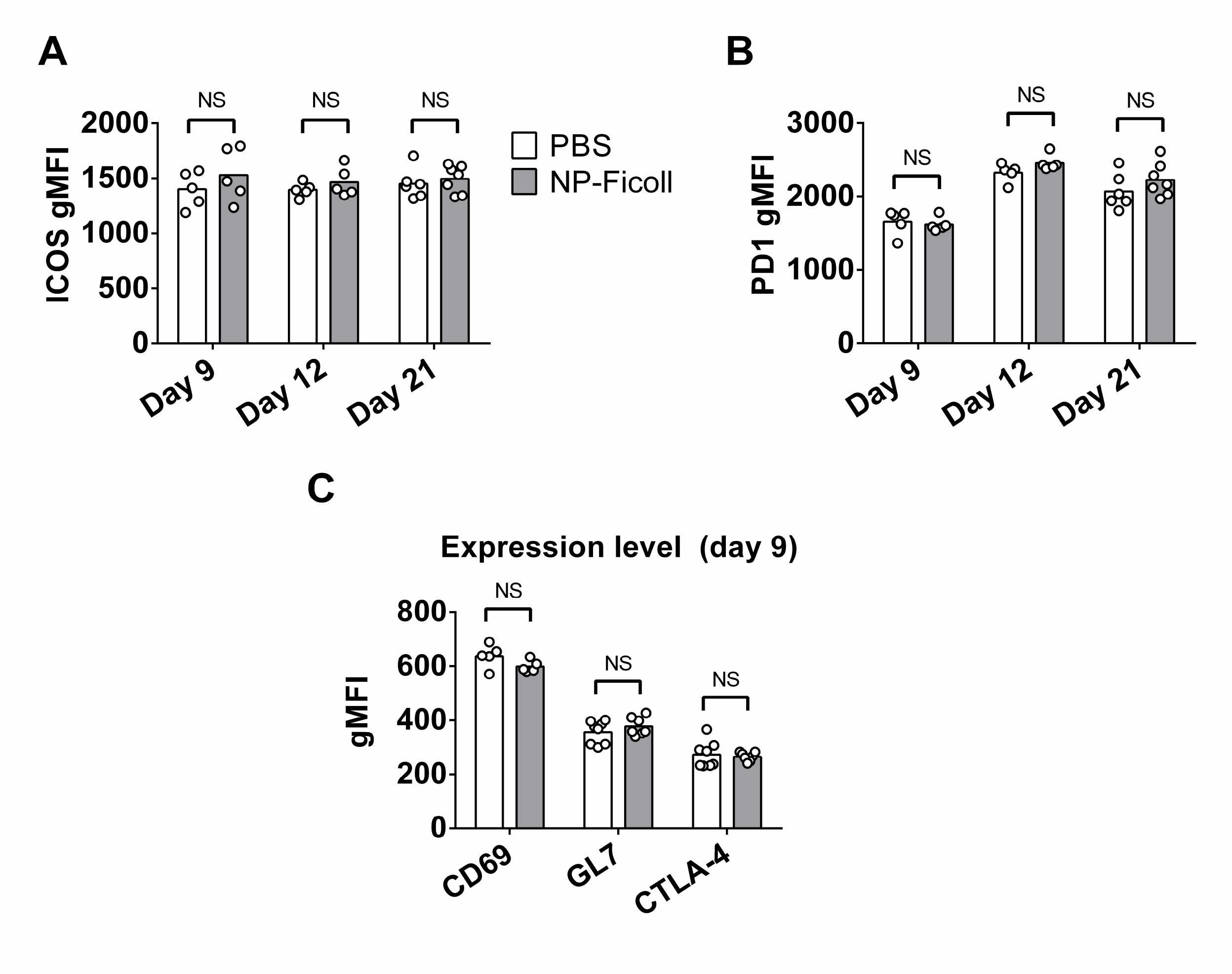

Figure S3. T follicular activation status after NP-Ficoll treatment, related to Figure 3.
(A-C) Mice were immunized and treated as in Figure 2A. T follicular cell $CD4^{+}$, $CD19^{-}$, $CXCR5^{hi}$, $PD1^{hi}$ expression level of various activation markers at indicated time points.
Bars represents mean; NS, not significant unpaired Welch's *t* test.

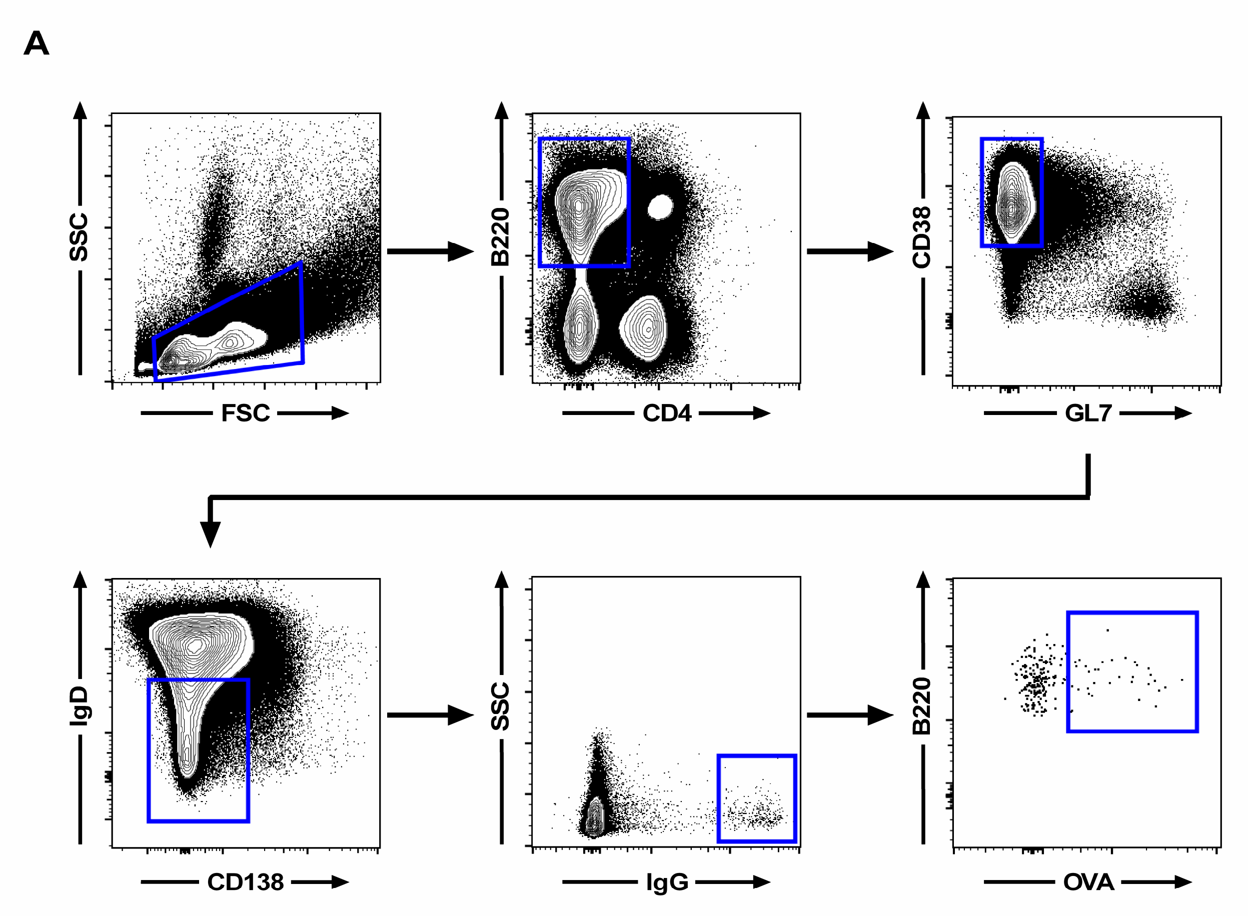

Fig. S4 OVA-specific memory B cell gating, related to Figure 4.
Mice were immunized and treated as in Figure 2A
(A) Gating strategy for OVA+ memory B cells.
