# Supplementary material for: Targeted Elimination of Immunodominant B Cells Drives the Germinal Center Reaction toward Subdominant Epitopes
Source: Cell Rep. Author manuscript; Available in PMC 2018 Jan 17. (PMC5771444; doi:10.1016/j.celrep.2017.12.014)
Supplement: 2 [file NIHMS931302-supplement-2.pdf]

Cell Reports

Report

Targeted Elimination of Immunodominant B Cells Drives the Germinal Center Reaction toward Subdominant Epitopes

Graphical Abstract

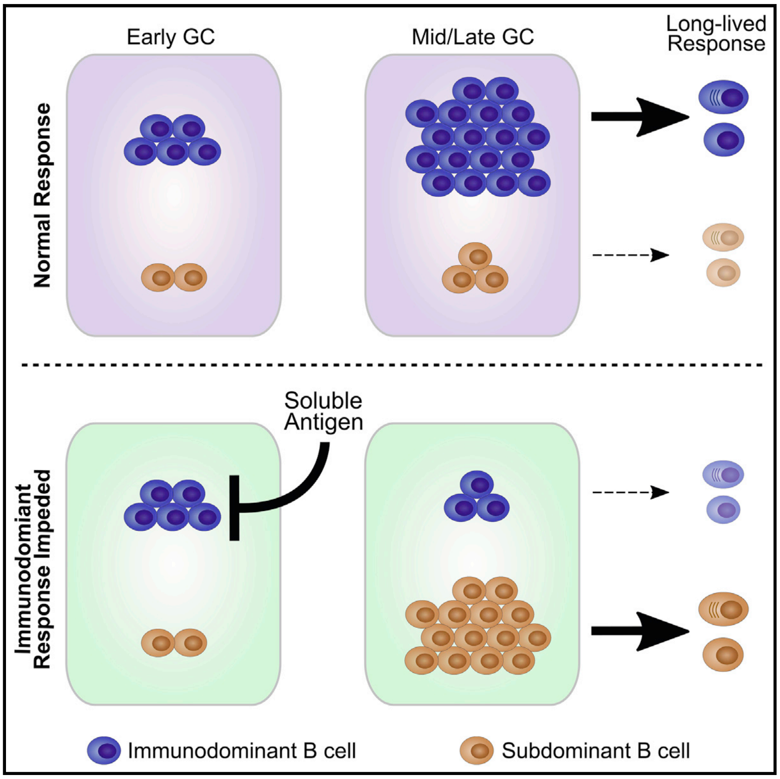

Highlights

- Soluble antigen can be used to selectively eliminate immunodominant GC B cells
- In the absence of immunodominant B cells, subdominant B cells overtake the GC response
- Suppression of immunodominant B cells improves subdominant long-lived humoral response

Authors

Murillo Silva, Thao H. Nguyen, Phaethon Philbrook, ..., Robert K. Abbott, Garnett Kelsoe, Michail V. Sitkovsky

Correspondence

silva.mu@gmail.com (M.S.),
m.sitkovsky@northeastern.edu (M.V.S.)

In Brief

Interclonal competition inhibits full participation of subdominant B cells in the germinal center (GC). Silva et al. demonstrate that selective elimination of immunodominant B cells during an active GC response allows subdominant B cells to expand unimpeded. Without competition, these subdominant cells generate an improved long-lived humoral response.

Silva et al., 2017, Cell Reports *21*, 3672–3680
December 26, 2017 © 2017 The Authors.
https://doi.org/10.1016/j.celrep.2017.12.014

OPEN ACCESS
CellPress
Cell Reports
Report

Targeted Elimination of Immunodominant B Cells Drives the Germinal Center Reaction toward Subdominant Epitopes

Murillo Silva,[1,*] Thao H. Nguyen,[1] Phaethon Philbrook,[1] Matthew Chu,[1] Olivia Sears,[1] Stephen Hatfield,[1] Robert K. Abbott,[1] Garnett Kelsoe,[2] and Michail V. Sitkovsky[1,3,*]
[1]New England Inflammation and Tissue Protection Institute, Northeastern University, Boston, MA 02115, USA
[2]Department of Immunology and Human Vaccine Institute, Duke University, Durham, NC 27710, USA
[3]Lead Contact
*Correspondence: silva.mu@gmail.com (M.S.), m.sitkovsky@northeastern.edu (M.V.S.)
https://doi.org/10.1016/j.celrep.2017.12.014

SUMMARY

Rapidly evolving pathogens such as HIV or influenza can quickly mutate their antigenic profiles, reducing the efficacy of conventional vaccines. Despite this challenge, functionally required epitopes are highly conserved among heterologous viral strains and represent a key vulnerability that could be targeted during vaccine development. As the antigenicity of these conserved epitopes is frequently subdominant, there is a critical need for innovative vaccination strategies designed to target these neutralizing epitopes. Here, we immunized mice with antigens containing discrete immunodominant and subdominant moieties and show that treatment with soluble heterologous antigen bearing only the immunodominant epitope selectively suppresses these germinal center (GC) B cells. By exploiting this intrinsic tolerance mechanism, we promote the expansion of subdominant B cells in the GC and the subsequent long-lived components of the humoral response. We propose that this strategy may be applied to elicit preferential expansion of subdominant B cells that recognize weakly immunogenic epitopes on microbial pathogens.

INTRODUCTION

The majority of approved vaccines function through the induction of long-lived neutralizing antibody (Ab) responses (Plotkin, 2010). Applying conventional vaccination strategies to viruses such as HIV or influenza, however, has not been effective at generating long-term protection against viral mutants that arise under immune selection (Hangartner et al., 2006; Haynes et al., 2012). Nevertheless, the fact that a small fraction of individuals can develop potent broadly neutralizing antibodies (bNAbs) after natural infection suggests that developing an effective vaccination strategy against these viruses should be physiologically possible (Johnston and Fauci, 2011). Immunodominance appears to be an important factor in preventing the generation of long-term protective immunity against elusive pathogens (Havenar-Daughton et al., 2017; Victora and Wilson, 2015). Strategies designed to overcome this obstacle have been largely focused on promoting activation of predicted bNAb B cell precursors by priming with engineered germline targeted immunogens (Escolano et al., 2016; Jardine et al., 2013; McGuire et al., 2013; Steichen et al., 2016) or increasing the overall breadth of the B cell response through the use of potent adjuvants such as MF59 (Khurana et al., 2010). Investigations into novel strategies that actively manipulate the germinal center (GC) selection process have not been well studied, however, and could provide an effective means to focus the B cell response toward desired epitopes.

The micro-anatomical structure of the GC is vital to the development of high-affinity antibodies (Eisen, 2014). In this location, B cell survival and expansion are regulated based on B cell receptor (BCR) affinity toward a particular antigen. Stochastic somatic hypermutation (SHM) of immunoglobulin genes, along with iterative cycles of clonal selection, drives an increase in average Ab affinity over the course of an immune response (Victora and Nussenzweig, 2012). Moreover, the GC is a major source of long-lived plasma cells and memory B cells, both critical to an effective vaccine response (Weisel and Shlomchik, 2017).

A T cell-based selection mechanism is, at least in part, responsible for regulating initial B cell entry and subsequent selection in the GC (Schwickert et al., 2011; Victora et al., 2010). This selection process predominately favors the entry of high-affinity clones, which are able to capture large amounts of antigen and display high densities of peptide-MHC II to a limited number of cognate T follicular helper (Tfh) cells. Although this competitive selection process is required for affinity maturation, it likely limits the diversity of B cell clones that can participate in the GC reaction (Dal Porto et al., 2002) and skews the immune response toward immunodominant epitopes (Havenar-Daughton et al., 2016).

As demonstrated by several laboratories, the administration of soluble antigen during an active GC response is highly effective at inducing antigen-specific B cells to undergo apoptosis (Chan et al., 2012; Han et al., 1995; Pulendran et al., 1995; Shokat and Goodnow, 1995; Victora et al., 2010). We hypothesized that we could exploit this intrinsic GC B cell tolerance mechanism to abrogate an immunodominant B cell response and to provide a survival advantage to the remaining subdominant B cell clones.

 © 2017 The Authors.
This is an open access article under the CC BY license (http://creativecommons.org/licenses/by/4.0/).

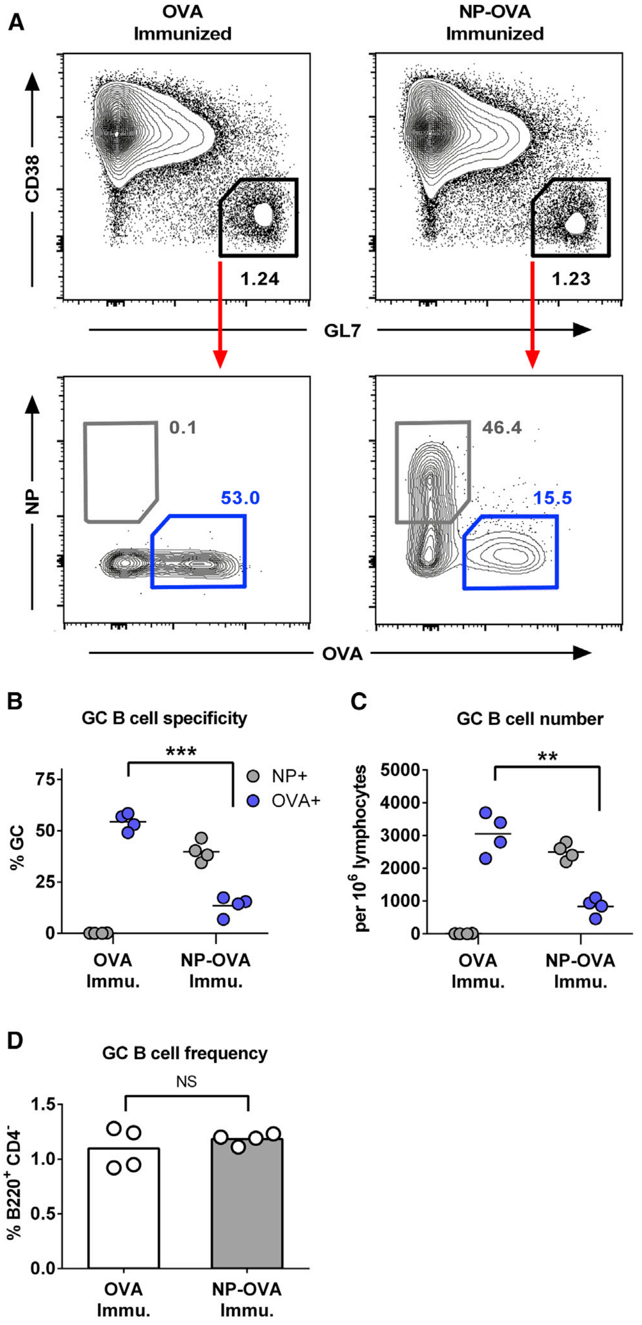

Figure 1. GC Competition Drives the B Cell Response toward Immunodominant Epitopes

C57BL/6J mice were immunized i.p. with 10 μg of either NP_4-OVA or OVA in precipitated alum. Spleens were dissected 12 days after immunization.

(A) Gating strategy for antigen-specific GC B cells ($B220^+$, $CD4^-$, $CD38^{lo}$, $GL7^+$, OVA^+/NP^-, or OVA^-/NP^+) and representative plots from each group.

(B and C) Quantification of antigen-specific B cell frequency (B) and cell count (C).

(D) GC B cell frequency after each immunization.

Cell counts are normalized to 10^6 lymphocytes. Bars represent mean; NS, not significant; $^{**}p < 0.01$, $^{***}p < 0.001$, unpaired Welch's t test. See also Figure S1.

Here, we immunized mice with the classical antigen 4-hydroxy-3-nitrophenylacetyl (NP)-ovalbumin (OVA) and show that soluble antigen containing only the dominant NP epitope (NP-Ficoll) can be administered to selectively target NP-specific GC B cells to be eliminated from the GC response. We found that this process allowed subdominant OVA-specific cells to expand and overtake the GC reaction. These otherwise-repressed cells generated an effective humoral response as seen by more abundant long-lived plasma cells, memory B cells, and increased Ab response. We propose that this strategy may be applied to elusive pathogens to direct the GC response toward specific epitopes of interest and elicit the preferential expansion of subdominant B cells that may be precursors to broadly neutralizing B cell clones.

RESULTS

NP-OVA As a Model for Interclonal Competition in the GC

NP-OVA contains two discrete antigenic moieties, the NP epitope and the polyepitopic OVA carrier protein, for which an antigen-specific GC response can be easily analyzed by fluorescently conjugated NP-phycoerytherin or OVA-Alexa Fluor 647 (Figure 1A). For our studies, we found that immunization with NP-OVA consistently generated a GC response where NP-specific cells comprised a 3-fold greater proportion of the GC as opposed to OVA (40 versus 14%, respectively; Figures 1A–1C). To test whether interclonal competition affects the participation of subdominant cells in our assay, we immunized C57BL/6J mice intraperitoneally (i.p.) with 10 μg of either NP-OVA or unconjugated OVA in precipitated alum. The mice were sacrificed 12 days after immunization, and spleens were harvested for analysis by flow cytometry. Both OVA- and NP-OVA-immunized groups generated comparable GC responses (Figures 1A and 1D) as well as T follicular and T follicular regulatory (Tfr) responses (Figures S1A and S1B, respectively). As expected, the presence of the immunodominant NP epitope significantly reduced the proportion of OVA-specific cells (14% NP-OVA immunized versus 55% in OVA immunized; Figures 1A–1C). Serum anti-OVA IgG Ab response was also reduced by the presence of the NP epitope (Figure S1C).

Consistent with recent reports that GCs are capable of maintaining a considerable level of clonal diversity (Kuraoka et al., 2016; Tas et al., 2016), we show that, despite the dominance of NP^+ cells, subdominant OVA^+ B cells still comprise a substantial portion of the GC during the immune response. Notwithstanding, these data demonstrate that interclonal competition reduces the proportion of subdominant B cell clones that participate in the GC reaction. In addition, it shows that, absent this competition, OVA-specific GC B cells are intrinsically capable of populating a large percentage (>50%) of the GC.

Soluble Antigen Treatment Reduces the Number of Immunodominant B Cells in GCs and Favors the Expansion of Subdominant Cells

Next, we asked whether eliminating immunodominant B cells during an active GC reaction could shift the response toward subdominant epitopes. We hypothesized that soluble antigen administration containing only the dominant NP epitope

(NP-Ficoll) would provide an effective way to selectively eliminate NP-specific GC B cells and relieve the selective pressure against OVA-specific cells. Two groups of mice were immunized with 10 μg of NP-OVA, and then treated with daily intravenous (i.v.) injections of either NP-Ficoll or PBS during the early GC response (days 6–8; Figure 2A). The effect of NP-Ficoll on GC B cell frequency and specificity was assessed 9, 12, and 21 days after immunization (1, 4, and 13 days after NP-Ficoll treatment). As expected, NP-specific GC B cells along with total GC B cell frequency were significantly reduced shortly following soluble antigen administration indicating GC B cell apoptosis (day 9; Figures 2B, 2C, S2A, and S2B). Consistent with previous reports (Han et al., 1995; Pulendran et al., 1995), this effect was observed to be antigen specific as NP-Ficoll administration to mice immunized with unconjugated OVA did not affect either total GC or OVA-specific B cell counts (Figures S2E and S2F). Additionally, this effect cannot be attributed to masking of the NP-specific BCR by NP-Ficoll as the prototypical NP-specific, λ1 light-chain-positive, B cells are also reduced following treatment (Figures S2C and S2D) (Jacob et al., 1993). Although the total GC frequency quickly recovered to control levels by day 12, the NP response, as measured by percent GC and cell number, was persistently reduced throughout the remainder of the GC response (through day 21; Figures 2D and 2E). In contrast, NP-Ficoll did not affect subdominant OVA-specific GC cell numbers; therefore, they encompassed a greater proportion of the smaller GC on day 9 (Figures 2D–2F). Importantly, as the GC response progressed, OVA^+ cells expanded significantly after elimination of NP^+ cells. Three days after the final soluble dose (day 12), OVA^+ cells increased by approximately 2-fold, as shown by both GC percentage and cell number, and remained elevated until the conclusion of the study (day 21; Figures 2D and 2F). Taken together, these data show that reducing interclonal competition during the early GC response significantly diminishes the selective pressure imposed on subdominant B cells and allows these cells to expand and encompass a greater proportion of the GC for the remainder of the response.

As the GC response to haptens can be an oversimplified representation of events occurring following protein immunization or microbial infection (Kuraoka et al., 2016; Tas et al., 2016), we next sought to validate these results using a more relevant complex antigen. To accomplish this while maintaining the ability to analyze discrete B cell specificities, we developed a protein-protein conjugate of CRM197 (non-toxic mutant of diphtheria toxin) and OVA at a 1:1 molar ratio to utilize as our immunizing antigen. Compared to mice immunized with unconjugated CRM197, CRM-OVA-immunized mice had a substantially weaker response to CRM, indicating that the OVA domain is immunodominant in this setting (Figures 2G, left; Figures S2I and S2J). We next asked whether eliminating immunodominant B cells during an active GC reaction could shift the response toward the subdominant CRM domain similarly to what had been observed in our previous experiments. Two groups of mice were immunized with 1 μg of CRM-OVA, and then treated with one i.p. injection of either 4 mg of OVA or PBS on day 7 post-immunization. The effect of soluble OVA on GC B cell frequency and specificity was assessed on day 12. As expected, mice treated with soluble OVA had a significant reduction in OVA-specific GC B cells (Figures 2G and S2H). Concurrently, OVA-treated mice had a significant increase in CRM-specific cells as assessed by both percent GC as well as cell count (Figure 2H). As with the previous experiments, total GC quantity was not affected at this time point (Figure S2G). These data show that the soluble antigen strategy may be utilized to skew the response to specific domains within a protein antigen and could prove useful in guiding the humoral response toward sought-after, conserved regions of microbial proteins.

NP-Ficoll Treatment Shifts OVA^+ GC B Cell Population to Dark Zone

As T follicular cells are critical to the development and maintenance of the GC reaction (Nutt and Tarlinton, 2011), we investigated how soluble antigen affects this population. We found no major perturbations in total T follicular and Tfr frequency in NP-Ficoll-treated mice (Figures 3A–3C). Their activation status (as measured by ICOS, PD1, CD69, GL7, and CTLA4 expression) also appears equivalent to control mice at all time points tested (Figure S3). As the GC B cell frequency is reduced by 48% shortly after soluble treatment (day 9; Figures 2B and 2C), the largely unchanged T follicular population effectively creates an environment with significantly increased T follicular-to-GC B cell ratio (Figure 3D). T follicular cell help has been shown to govern B cell residence time in the dark zone (DZ) of GCs and are thought to ultimately control their proliferative capacity (Gitlin et al., 2014, 2015). Consistent with previous reports, our data suggest that the increased level of T cell help provided in soluble antigen-treated mice shifted the remaining OVA-specific cells to the DZ of the GC (Victora et al., 2010) and supported the expansion of this population (Figures 2F, 3E, and 3F). Moreover, OVA-specific GC B cells from NP-Ficoll-treated mice had increased mammalian target of rapamycin (mTOR) activity (as measured by phosphorylated ribosomal protein S6; Figures 3G and 3H), which is indicative that these cells received stronger T cell help and were primed to undergo sustained proliferation in the DZ (Ersching et al., 2017). The data show that by eliminating the dominant B cell response, NP-Ficoll treatment appears to create an environment that is supportive for the remaining OVA^+ GC B cells and suggests how these subdominant cells quickly expand following treatment.

Attenuating GC Competition Increases Subdominant Long-Lived Plasma Cell, Memory B Cell, and Ab Production

As the GC reaction is the major source of a long-lived humoral response (Good-Jacobson and Shlomchik, 2010), we next investigated whether the GC specificity changes imposed by our treatment would translate to downstream functional effects. After 21 days following immunization, bone marrow (BM) cells were isolated and cultured to assess the extent of NP^+ and OVA^+ plasma cell (PC) formation. Enzyme-linked immunospot (ELISPOT) analysis demonstrated that elimination of NP^+ cells during the early GC response translates to a reduction in NP-specific PC formation (Figure 4A). Importantly, the frequency of OVA-specific PC in BM was considerably higher following NP-Ficoll treatment (Figure 4B), in excellent correlation with

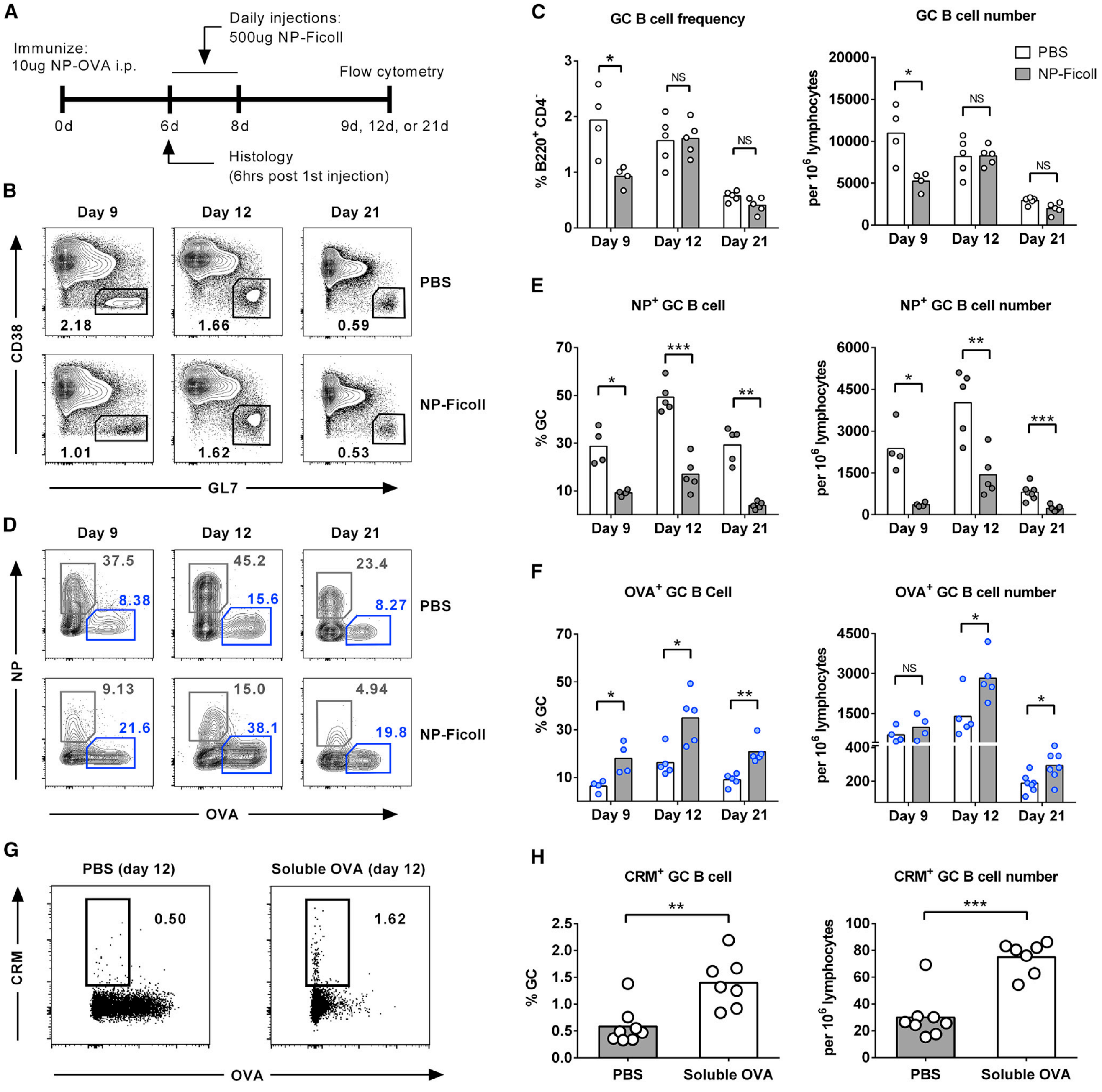

Figure 2. Soluble Antigen Treatment Reduces the Number of Immunodominant B Cells in GCs and Favors the Expansion of Subdominant Cells

(A) Schematic outline of experimental approach for (A–F). Mice were immunized i.p. with 10 μg of NP_4-OVA in precipitated alum, and then treated with soluble NP-Ficoll or PBS on days 6 through 8. Spleens were dissected 9, 12, or 21 days after immunization.

(B and C) Representative plots of GC frequency ($B220^+$, $CD4^-$, $GL7^+$, $CD38^{lo}$) (B) and quantification (C).

(D) Representative gating for antigen-specific GC B cells ($B220^+$, $GL7^+$, $CD4^-$, $CD38^{lo}$, and OVA^+/NP^-, or OVA^-/NP^+).

(E and F) Quantification of NP-specific (E) and OVA-specific (F) GC B cell frequency and cell number.

(G and H) Mice were immunized i.p. with 1 μg of CRM-OVA, and then treated with soluble OVA or PBS on day 7. Spleens were dissected 12 days after immunization. Representative plots of antigen-specific GC B cells (G) and quantification of CRM^+ cells (H).

Cell counts are normalized to 10^6 lymphocytes. Bars represent mean; NS, not significant; *p < 0.05, **p < 0.01, ***p < 0.001, Welch's t test. See also Figure S2.

our observation that the GC response was redirected toward the OVA epitope. Consistent with these results, the long-term IgG Ab response was significantly affected by the manipulations imposed on the early GC reaction. OVA-specific Ab in NP-Ficoll-treated mice were markedly higher from day 35 through the remainder of our assay (day 63; Figure 4D). The OVA Ab

OPEN
ACCESS
CellPress

response in treated mice peaked long after the control group (day 49 versus day 21), corroborating the increase in BM PC counts. Conversely, the NP-specific Ab response was significantly decreased during the late humoral response (day 35 through day 63; Figure 4C). This late difference in Ab response is consistent with previous reports demonstrating that long-lived PCs are primarily generated during the late stages of the GC response (Weisel et al., 2016) and is also indicative that soluble antigen treatment is predominantly affecting the GC response but not the extrafollicular response.

As the goal of any vaccine is to generate a memory response that is able to quickly react to the secondary challenge of an invading pathogen (Weisel and Shlomchik, 2017), we analyzed how our treatment affected the development of this population. In agreement with our GC and PC data, NP-Ficoll-treated mice developed significantly higher IgG1-switched OVA$^+$ memory phenotype B cells at both time points tested (Figures 4E, 4F, and S4). Additionally, NP-Ficoll-treated mice showed a robust increase in α-OVA Ab production upon antigen challenge 21 weeks after primary immunization, while nearly all control mice failed to respond (Figure 4G). Recent reports have shown that low-affinity B cells are preferentially selected for memory B cell differentiation (Shinnakasu et al., 2016). We view such a mechanism as consistent with our data. In an untreated setting, a small portion of OVA-specific GC B cells are capable of surviving despite the presence of immunodominant NP-specific cells. We believe that, in order to remain viable, these OVA$^+$ cells are likely to be of relatively high affinity. However, once the immunodominant cells are eliminated by soluble antigen treatment, it is possible that the more permissive environment tolerates the survival of lower-affinity OVA-specific GC B cells and could explain the observed increase in OVA$^+$ memory B cell differentiation as well as Ab recall response in our studies. Given these findings, we conclude that attenuating the immunodominant response during the early GC reaction promotes a long-lived humoral response against subdominant epitopes.

DISCUSSION

Viral envelope proteins are primary targets for neutralizing Abs, but mutations in these structural proteins regularly enable viral escape from nascent immunity (Skehel and Wiley, 2000; Ward and Wilson, 2015). Influenza viruses acquire these escape mutations seasonally in populations, while mutations in HIV-1 arise in chronically infected individuals (Hangartner et al., 2006). Mutational escape from the immune response to maintain viral fitness may be the driving force for the evolution of viral envelope proteins that contain variable regions, which have little impact on

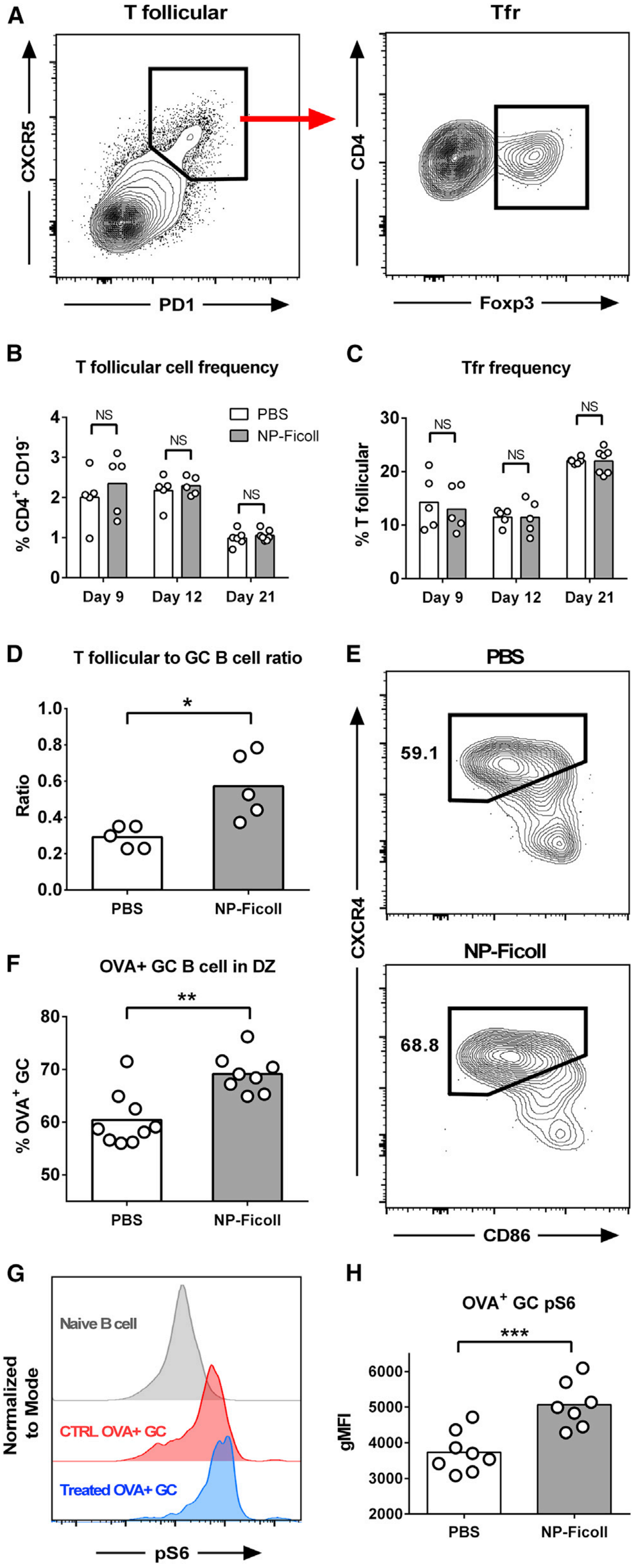

Figure 3. NP-Ficoll Treatment Shifts OVA$^+$ GC B Cell Population to DZ

Mice were immunized and treated as in Figure 2A.

(A) Gating strategy for total T follicular or Tfr cells (CD4$^+$, CD19$^-$, CXCR5^{hi}, PD1^{hi} and CD4$^+$, CD19$^-$, CXCR5^{hi}, PD1^{hi}, Foxp3$^{-/+}$).

(B and C) Quantification of T follicular (B) and Tfr cells (C).

(D) Ratio of T follicular to GC B cell after NP-Ficoll treatment on day 9.

(E and F) Representative OVA-specific GC dark zone (DZ) gate (B220$^+$, GL7$^+$, CD4$^-$, CD38^{lo}, OVA$^+$, CxCr4^{hi}, CD86^{lo}) (E) and quantification (F) on day 9.

(G and H) Representative staining intensity of pS6 (G) and quantification (H).

Bars represent mean; NS, not significant; *p < 0.05, **p < 0.01, unpaired Welch's t test. See also Figure S3.

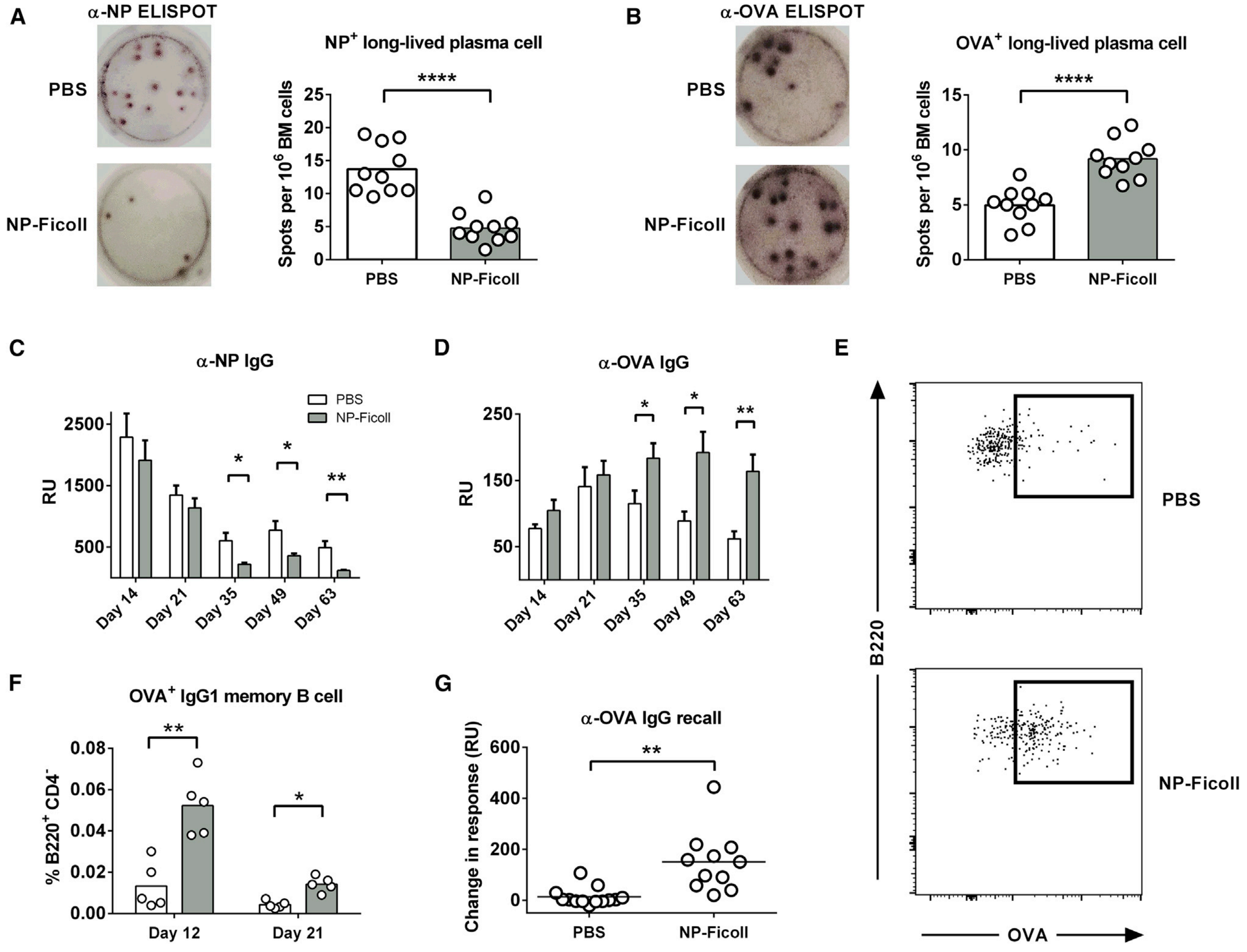

Figure 4. Attenuating GC Competition Increases Subdominant Long-Lived PC, Memory B Cell, and Ab Production

Mice were immunized and treated as in Figure 2A.

(A and B) NP-specific (A) and OVA-specific (B) ELISPOTs obtained from BM 21 days after primary immunization. Spots are representative of three replicate wells per mice, 10 mice per group.

(C and D) Serum anti-NP (C) and anti-OVA Ab (D) obtained at various time points. Bar represents mean ± SEM, n = 7 or 8 per group.

(E and F) Representative flow plots of IgG1$^+$ OVA-specific memory B cells (B220$^+$, CD4$^-$, CD38$^+$, GL7$^-$, CD138$^-$, IgD$^{lo/-}$, IgG1$^+$, OVA$^+$) (E) and quantification (F).

(G) α-OVA Ab recall response. Mice were allowed to rest for 21 weeks after immunization and were then challenged with 10 μg of NP-OVA without adjuvant. Data are pooled from two independent experiments representing change in Ab response from 1 day before challenge to 8 days post-challenge for individual mice. Bars represent mean; *p < 0.05, **p < 0.01, ****p < 0.0001, Welch's t test. See also Figure S4.

viral function, and conserved regions, which are necessary for virus transmission and replication (Corti and Lanzavecchia, 2013). Epitopes within these conserved, functional regions are targets for bNAbs and viruses employ various strategies to obscure these conserved domains from immune surveillance (Townsley et al., 2015; Wei et al., 2003). The consequence is that conserved, functional domains are often weakly immunogenic or immunologically subdominant and that standard immunization protocols often do not elicit robust, high-affinity Ab responses to these cryptic domains (Kelsoe et al., 2014).

Indeed, the immunodominance of non-conserved virus epitopes appears to be a major challenge in current efforts to develop effective HIV and influenza vaccines (Havenar-Daughton et al., 2017; Victora and Wilson, 2015). Evidence from several HIV bNAb producers shows that reverted germline B cell clones bind only weakly to broadly neutralizing epitopes of HIV (Hoot et al., 2013; Liao et al., 2013; Xiao et al., 2009) and raises the possibility that many of these cells fail to activate following traditional vaccination approaches. A promising strategy designed to address this obstacle has been to specifically target bNAb precursor B cell clones by priming with engineered immunogens that more avidly bind these cells (Briney et al., 2016; Dosenovic et al., 2015; Escolano et al., 2016; Steichen et al., 2016). Although this strategy proved to be effective in Ig-knockin mice, competition from other responding clones may pose an obstacle in genetically unmodified organisms.

The phenomena of "original antigenic sin" (Davenport et al., 1953) could be an additional hindrance to broadly neutralizing influenza vaccine development. Preexisting immunological history has been shown to strongly skew secondary humoral responses to structurally related epitopes (Andrews et al., 2015) and could inhibit the recruitment and activation of desired subdominant B cell clones. In order to address these significant challenges, the development of vaccination strategies that complement and build upon the substantial progress already made in the field of immunogen design will likely be necessary.

Although our studies were mainly focused on overall GC specificity, it remains to be tested whether this treatment regimen affects the frequency of SHM and the rate of affinity maturation of B cells responding to subdominant epitopes. Additionally, it is important to note that while systemic treatment with large doses of soluble antigen may not be amenable for direct translation to human vaccine strategies, our experiments provide a proof of principle that we hope can be utilized for future vaccine development. An important next step for the concepts presented here will be to implement methods that can deliver soluble antigen specifically to the site where it is needed. A suitable method may be to utilize lymph-node (LN)-targeting strategies such as the "albumin hitchhiking" approach that has been shown to increase trafficking and accumulation of antigen to LN by more than 10-fold (Liu et al., 2014). Targeted delivery systems such as this could theoretically be coupled to cytotoxic or inhibitory agents to reduce the amount of antigen required to induce immunodominant GC B cell apoptosis/inhibition. Optimization of the overall strategy will facilitate future investigations on whether skewing the GC response will in fact be advantageous in a prophylactic vaccine setting.

In summary, we propose a fundamentally different approach to vaccination that actively manipulates the GC selection process and is capable of directing the response toward otherwise subdominant epitopes. Importantly, these effects proved to be functionally relevant as greater GC participation translated into increased subdominant Ab production as well as long-lived plasma and memory B cell generation. Overall, this work demonstrates the key role that immunodominant epitopes play in shaping the GC response. Innovative strategies that can overcome this obstacle could prove to be an effective means to expand the desired population of bNAb precursor B cell clones and may lead to the development of new vaccination strategies.

EXPERIMENTAL PROCEDURES

Mice, Immunizations, and Treatments

Animal work was in accordance with Institutional Animal Care and Use Committee at Northeastern University. C57BL/6J mice were purchased from The Jackson Laboratory (Bar Harbor, ME) and held under specific-pathogen-free conditions. Age- and sex-matched mice between 7 and 10 weeks of age were used for all experiments. Four to ten mice per group were used in each analytical experiment. NP-OVA was conjugated in-house at a molar ratio of 4:1 NP:OVA. NP-OSu was purchased from Biosearch Technologies (N-1010-100), and OVA was obtained from Sigma-Aldrich (A5503). Unconjugated NP hapten was removed using Amicon Ultra 30K centrifugal filter units (Millipore). CRM197 was purchased from Scarab Genomics and was conjugated to OVA at a 1:1 molar ratio using Pierce Controlled Protein-Protein Crosslinking kit (Thermo Scientific) following the manufacturer's instructions. Mice were immunized with 10 μg of NP-OVA i.p. in precipitated alum as described by Cain et al. (2013). NP-Ficoll was purchased from Biosearch Technologies (F-1420-100). Mice were treated with daily i.v. injections of 500 μg of NP_{18}-Ficoll or NP_{30}-Ficoll from days 6 to 8 following immunization. For memory recall experiments, mice were immunized with NP-OVA and treated with NP-Ficoll as stated above and allowed to rest for 21 weeks. 10 μg of NP-OVA without adjuvant was administered i.v., and serum was collected 8 days later for analysis. In some experiments, mice were immunized with 1 μg of CRM-OVA in alum i.p., and then treated with 4 mg of soluble OVA on day 7 along with 150 μg of isoproterenol (Millipore) as previously described (Han et al., 1995).

Flow Cytometry

Spleens were forced through a 70-μm strainer into PBS supplemented with 5% fetal calf serum (FCS). Red blood cells (RBCs) were lysed with ACK buffer (Gilbco) followed by 10-min incubation with 1 μg/mL Fc Block (24G2; BD Biosciences), and then stained for 30 min at 4°C with the following antibodies acquired from Biolegend or BD Biosciences: B220 (RA3-6B2), CD138 (281-2), CD19 (ID3), CD38 (90), CD4 (RM4-5), CD86 (GL-1), CXCR5 (2G8), CXCR4 (2B11), Foxp3 (MF-23), GL-7 (GL7), ICOS (7E.17G9), IgD (11-26c.2a), IgG1 (A85-1), and PD-1 (J43). Antigen-specific GC responses were detected with Alexa Fluor 647-tagged OVA (Thermo Fisher), NP-phycoerythrin (PE) (Biosearch Technologies), or with FITC-tagged CRM (made in house) amplified by biotinylated α-FITC (Biolegend) and BB515 streptavidin (BD Biosciences). Phospho-S6 (pS6) staining was performed as described by Ersching et al. (2017). For intracellular staining, samples were fixed and permeabilized prior to staining (Foxp3/transcription factor buffer set; eBioscience). Acquisition was conducted on a Cytek DxP11 FACSCalibur and analyzed on FlowJo X (Tree Star).

Immunohistochemistry

Spleens from immunized mice were frozen in Tissue-Tek compound in a liquid nitrogen-cooled bath of 2-methylbutane. The 5-μm sections were cut on a cryostat, air-dried, and then fixed in ice-cold acetone for 10 min. Sections were rehydrated (0.5% BSA, 0.1% Tween 20 in PBS), FC-blocked, and stained with TUNEL apoptosis kit (Invitrogen; C10619) following the manufacturer's instructions. Sections were then stained with PNA-FITC (Vector Labs) and B220 (RA3-6B2) for 1 hr at room temperature (RT). Images were acquired on a Zeiss LSM 710 microscope, and ImageJ/Fiji was used for analysis. Briefly, mask files were created for GCs (PNA^+, $B220^+$ area) and apoptotic nuclei ($TUNEL^+$). Relative apoptosis was quantified based on percent $TUNEL^+$ area within individual GC area.

ELISA and ELISPOT

For ELISA, 96-well plates (2595; Costar) were coated with 2 μg/mL NP_{30}-BSA or 10 μg/mL OVA (Biosearch Technologies and Sigma-Aldrich) in PBS overnight. Plates were washed twice (0.5% BSA, 0.1% Tween 20 in PBS), blocked for 1 hr (0.5% BSA in PBS), and washed twice, and serially diluted samples were added for 1 hr at RT. Plates were washed three times and horseradish peroxidase (HRP)-conjugated detection Abs for IgG (Bethyl Laboratories) were added for 1 hr, washed three times, and tetramethylbenzidine substrate was added (BD Biosciences). Reaction was stopped using 2 N H_2SO_4 and read at 450 nm. Ab quantification was calculated based on NP- or OVA-specific monoclonal standards (H33lγ1 or OVA-14) and reported as relative units (RU). For ELISPOTs, Immobilon-P plates (Millipore) were coated overnight at 4°C with 2 μg/mL NP_{30}-BSA or 10 μg/mL OVA in PBS. Plates were washed twice then blocked for 2 hr. BM cells were isolated; RBCs were lysed with ACK, and then incubated for 3 hr at 37°C in RPMI. The plates were washed three times, and then incubated with alkaline phosphatase-conjugated Abs to IgG (SouthernBiotech). Plates were washed five times and developed using NBT reagent (Sigma-Aldrich).

Statistical Analyses

All statistical analysis was performed using GraphPad Prism. Statistical significance was determined using a two-tailed unpaired Welch's t test. p values of less than 0.05 were considered significant.

SUPPLEMENTAL INFORMATION

Supplemental Information includes four figures and can be found with this article online at https://doi.org/10.1016/j.celrep.2017.12.014.

ACKNOWLEDGMENTS

We thank Susan Ohman for careful editing of the manuscript. We thank Dr. Zupanc and Antonia Vitalo for use of their cryostat. We also thank Jason Chang for help with the Graphical Abstract. This work was supported in part by NIH Grants U19 AI 091693 and R01 CA 111985 to M.V.S. M.S. and M.V.S. have filed a patent based on this work.

AUTHOR CONTRIBUTIONS

M.S. developed the project, designed experiments, interpreted data, and wrote the manuscript. T.H.N. conducted experiments and edited the manuscript. P.P., M.C., O.S., and S.H. conducted experiments. M.V.S., G.K., and R.K.A. designed experiments, interpreted data, and edited the manuscript. All authors reviewed the manuscript and approved final submission.

DECLARATION OF INTERESTS

M.S. and M.V.S. have filed a patent based on this work.

Received: May 29, 2017
Revised: October 14, 2017
Accepted: December 1, 2017
Published: December 26, 2017

REFERENCES

Andrews, S.F., Huang, Y., Kaur, K., Popova, L.I., Ho, I.Y., Pauli, N.T., Henry Dunand, C.J., Taylor, W.M., Lim, S., Huang, M., et al. (2015). Immune history profoundly affects broadly protective B cell responses to influenza. Sci. Transl. Med. *7*, 316ra192.

Briney, B., Sok, D., Jardine, J.G., Kulp, D.W., Skog, P., Menis, S., Jacak, R., Kalyuzhniy, O., de Val, N., Sesterhenn, F., et al. (2016). Tailored immunogens direct affinity maturation toward HIV neutralizing antibodies. Cell *166*, 1459–1470.e11.

Cain, D.W., Sanders, S.E., Cunningham, M.M., and Kelsoe, G. (2013). Disparate adjuvant properties among three formulations of ‘‘alum.’’. Vaccine *31*, 653–660.

Chan, T.D., Wood, K., Hermes, J.R., Butt, D., Jolly, C.J., Basten, A., and Brink, R. (2012). Elimination of germinal-center-derived self-reactive B cells is governed by the location and concentration of self-antigen. Immunity *37*, 893–904.

Corti, D., and Lanzavecchia, A. (2013). Broadly neutralizing antiviral antibodies. Annu. Rev. Immunol. *31*, 705–742.

Dal Porto, J.M., Haberman, A.M., Kelsoe, G., and Shlomchik, M.J. (2002). Very low affinity B cells form germinal centers, become memory B cells, and participate in secondary immune responses when higher affinity competition is reduced. J. Exp. Med. *195*, 1215–1221.

Davenport, F.M., Hennessy, A.V., and Francis, T., Jr. (1953). Epidemiologic and immunologic significance of age distribution of antibody to antigenic variants of influenza virus. J. Exp. Med. *98*, 641–656.

Dosenovic, P., von Boehmer, L., Escolano, A., Jardine, J., Freund, N.T., Gitlin, A.D., McGuire, A.T., Kulp, D.W., Oliveira, T., Scharf, L., et al. (2015). Immunization for HIV-1 broadly neutralizing antibodies in human Ig knockin mice. Cell *161*, 1505–1515.

Eisen, H.N. (2014). Affinity enhancement of antibodies: how low-affinity antibodies produced early in immune responses are followed by high-affinity antibodies later and in memory B-cell responses. Cancer Immunol. Res. *2*, 381–392.

Ersching, J., Efeyan, A., Mesin, L., Jacobsen, J.T., Pasqual, G., Grabiner, B.C., Dominguez-Sola, D., Sabatini, D.M., and Victora, G.D. (2017). Germinal center selection and affinity maturation require dynamic regulation of mTORC1 kinase. Immunity *46*, 1045–1058.e6.

Escolano, A., Steichen, J.M., Dosenovic, P., Kulp, D.W., Golijanin, J., Sok, D., Freund, N.T., Gitlin, A.D., Oliveira, T., Araki, T., et al. (2016). Sequential immunization elicits broadly neutralizing anti-HIV-1 antibodies in Ig knockin mice. Cell *166*, 1445–1458.e12.

Gitlin, A.D., Shulman, Z., and Nussenzweig, M.C. (2014). Clonal selection in the germinal centre by regulated proliferation and hypermutation. Nature *509*, 637–640.

Gitlin, A.D., Mayer, C.T., Oliveira, T.Y., Shulman, Z., Jones, M.J., Koren, A., and Nussenzweig, M.C. (2015). HUMORAL IMMUNITY. T cell help controls the speed of the cell cycle in germinal center B cells. Science *349*, 643–646.

Good-Jacobson, K.L., and Shlomchik, M.J. (2010). Plasticity and heterogeneity in the generation of memory B cells and long-lived plasma cells: the influence of germinal center interactions and dynamics. J. Immunol. *185*, 3117–3125.

Han, S., Zheng, B., Dal Porto, J., and Kelsoe, G. (1995). In situ studies of the primary immune response to (4-hydroxy-3-nitrophenyl)acetyl. IV. Affinity-dependent, antigen-driven B cell apoptosis in germinal centers as a mechanism for maintaining self-tolerance. J. Exp. Med. *182*, 1635–1644.

Hangartner, L., Zinkernagel, R.M., and Hengartner, H. (2006). Antiviral antibody responses: the two extremes of a wide spectrum. Nat. Rev. Immunol. *6*, 231–243.

Havenar-Daughton, C., Carnathan, D.G., Torrents de la Peña, A., Pauthner, M., Briney, B., Reiss, S.M., Wood, J.S., Kaushik, K., van Gils, M.J., Rosales, S.L., et al. (2016). Direct probing of germinal center responses reveals immunological features and bottlenecks for neutralizing antibody responses to HIV Env trimer. Cell Rep. *17*, 2195–2209.

Havenar-Daughton, C., Lee, J.H., and Crotty, S. (2017). Tfh cells and HIV bnAbs, an immunodominance model of the HIV neutralizing antibody generation problem. Immunol. Rev. *275*, 49–61.

Haynes, B.F., Kelsoe, G., Harrison, S.C., and Kepler, T.B. (2012). B-cell-lineage immunogen design in vaccine development with HIV-1 as a case study. Nat. Biotechnol. *30*, 423–433.

Hoot, S., McGuire, A.T., Cohen, K.W., Strong, R.K., Hangartner, L., Klein, F., Diskin, R., Scheid, J.F., Sather, D.N., Burton, D.R., and Stamatatos, L. (2013). Recombinant HIV envelope proteins fail to engage germline versions of anti-CD4bs bNAbs. PLoS Pathog. *9*, e1003106.

Jacob, J., Przylepa, J., Miller, C., and Kelsoe, G. (1993). In situ studies of the primary immune response to (4-hydroxy-3-nitrophenyl)acetyl. III. The kinetics of V region mutation and selection in germinal center B cells. J. Exp. Med. *178*, 1293–1307.

Jardine, J., Julien, J.P., Menis, S., Ota, T., Kalyuzhniy, O., McGuire, A., Sok, D., Huang, P.S., MacPherson, S., Jones, M., et al. (2013). Rational HIV immunogen design to target specific germline B cell receptors. Science *340*, 711–716.

Johnston, M.I., and Fauci, A.S. (2011). HIV vaccine development—improving on natural immunity. N. Engl. J. Med. *365*, 873–875.

Kelsoe, G., Verkoczy, L., and Haynes, B.F. (2014). Immune system regulation in the induction of broadly neutralizing HIV-1 antibodies. Vaccines (Basel) *2*, 1–14.

Khurana, S., Chearwae, W., Castellino, F., Manischewitz, J., King, L.R., Honorkiewicz, A., Rock, M.T., Edwards, K.M., Del Giudice, G., Rappuoli, R., and Golding, H. (2010). Vaccines with MF59 adjuvant expand the antibody repertoire to target protective sites of pandemic avian H5N1 influenza virus. Sci. Transl. Med. *2*, 15ra5.

Kuraoka, M., Schmidt, A.G., Nojima, T., Feng, F., Watanabe, A., Kitamura, D., Harrison, S.C., Kepler, T.B., and Kelsoe, G. (2016). Complex antigens drive permissive clonal selection in germinal centers. Immunity *44*, 542–552.

Liao, H.X., Lynch, R., Zhou, T., Gao, F., Alam, S.M., Boyd, S.D., Fire, A.Z., Roskin, K.M., Schramm, C.A., Zhang, Z., et al.; NISC Comparative Sequencing Program (2013). Co-evolution of a broadly neutralizing HIV-1 antibody and founder virus. Nature *496*, 469–476.

Liu, H., Moynihan, K.D., Zheng, Y., Szeto, G.L., Li, A.V., Huang, B., Van Egeren, D.S., Park, C., and Irvine, D.J. (2014). Structure-based programming of lymph-node targeting in molecular vaccines. Nature *507*, 519–522.

McGuire, A.T., Hoot, S., Dreyer, A.M., Lippy, A., Stuart, A., Cohen, K.W., Jardine, J., Menis, S., Scheid, J.F., West, A.P., et al. (2013). Engineering HIV envelope protein to activate germline B cell receptors of broadly neutralizing anti-CD4 binding site antibodies. J. Exp. Med. *210*, 655–663.

Nutt, S.L., and Tarlinton, D.M. (2011). Germinal center B and follicular helper T cells: siblings, cousins or just good friends? Nat. Immunol. *12*, 472–477.

Plotkin, S.A. (2010). Correlates of protection induced by vaccination. Clin. Vaccine Immunol. *17*, 1055–1065.

Pulendran, B., Kannourakis, G., Nouri, S., Smith, K.G., and Nossal, G.J. (1995). Soluble antigen can cause enhanced apoptosis of germinal-centre B cells. Nature *375*, 331–334.

Schwickert, T.A., Victora, G.D., Fooksman, D.R., Kamphorst, A.O., Mugnier, M.R., Gitlin, A.D., Dustin, M.L., and Nussenzweig, M.C. (2011). A dynamic T cell-limited checkpoint regulates affinity-dependent B cell entry into the germinal center. J. Exp. Med. *208*, 1243–1252.

Shinnakasu, R., Inoue, T., Kometani, K., Moriyama, S., Adachi, Y., Nakayama, M., Takahashi, Y., Fukuyama, H., Okada, T., and Kurosaki, T. (2016). Regulated selection of germinal-center cells into the memory B cell compartment. Nat. Immunol. *17*, 861–869.

Shokat, K.M., and Goodnow, C.C. (1995). Antigen-induced B-cell death and elimination during germinal-centre immune responses. Nature *375*, 334–338.

Skehel, J.J., and Wiley, D.C. (2000). Receptor binding and membrane fusion in virus entry: the influenza hemagglutinin. Annu. Rev. Biochem. *69*, 531–569.

Steichen, J.M., Kulp, D.W., Tokatlian, T., Escolano, A., Dosenovic, P., Stanfield, R.L., McCoy, L.E., Ozorowski, G., Hu, X., Kalyuzhniy, O., et al. (2016). HIV vaccine design to target germline precursors of glycan-dependent broadly neutralizing antibodies. Immunity *45*, 483–496.

Tas, J.M., Mesin, L., Pasqual, G., Targ, S., Jacobsen, J.T., Mano, Y.M., Chen, C.S., Weill, J.C., Reynaud, C.A., Browne, E.P., et al. (2016). Visualizing antibody affinity maturation in germinal centers. Science *351*, 1048–1054.

Townsley, S., Li, Y., Kozyrev, Y., Cleveland, B., and Hu, S.L. (2015). Conserved role of an N-linked glycan on the surface antigen of human immunodeficiency virus type 1 modulating virus sensitivity to broadly neutralizing antibodies against the receptor and coreceptor binding sites. J. Virol. *90*, 829–841.

Victora, G.D., and Nussenzweig, M.C. (2012). Germinal centers. Annu. Rev. Immunol. *30*, 429–457.

Victora, G.D., and Wilson, P.C. (2015). Germinal center selection and the antibody response to influenza. Cell *163*, 545–548.

Victora, G.D., Schwickert, T.A., Fooksman, D.R., Kamphorst, A.O., Meyer-Hermann, M., Dustin, M.L., and Nussenzweig, M.C. (2010). Germinal center dynamics revealed by multiphoton microscopy with a photoactivatable fluorescent reporter. Cell *143*, 592–605.

Ward, A.B., and Wilson, I.A. (2015). Insights into the trimeric HIV-1 envelope glycoprotein structure. Trends Biochem. Sci. *40*, 101–107.

Wei, X., Decker, J.M., Wang, S., Hui, H., Kappes, J.C., Wu, X., Salazar-Gonzalez, J.F., Salazar, M.G., Kilby, J.M., Saag, M.S., et al. (2003). Antibody neutralization and escape by HIV-1. Nature *422*, 307–312.

Weisel, F., and Shlomchik, M. (2017). Memory B cells of mice and humans. Annu. Rev. Immunol. *35*, 255–284.

Weisel, F.J., Zuccarino-Catania, G.V., Chikina, M., and Shlomchik, M.J. (2016). A temporal switch in the germinal center determines differential output of memory B and plasma cells. Immunity *44*, 116–130.

Xiao, X., Chen, W., Feng, Y., Zhu, Z., Prabakaran, P., Wang, Y., Zhang, M.Y., Longo, N.S., and Dimitrov, D.S. (2009). Germline-like predecessors of broadly neutralizing antibodies lack measurable binding to HIV-1 envelope glycoproteins: implications for evasion of immune responses and design of vaccine immunogens. Biochem. Biophys. Res. Commun. *390*, 404–409.

Cell Reports, Volume *21*

Supplemental Information

Targeted Elimination of Immunodominant B Cells Drives the Germinal Center Reaction toward Subdominant Epitopes

Murillo Silva, Thao H. Nguyen, Phaethon Philbrook, Matthew Chu, Olivia Sears, Stephen Hatfield, Robert K. Abbott, Garnett Kelsoe, and Michail V. Sitkovsky

Supplemental Figures:

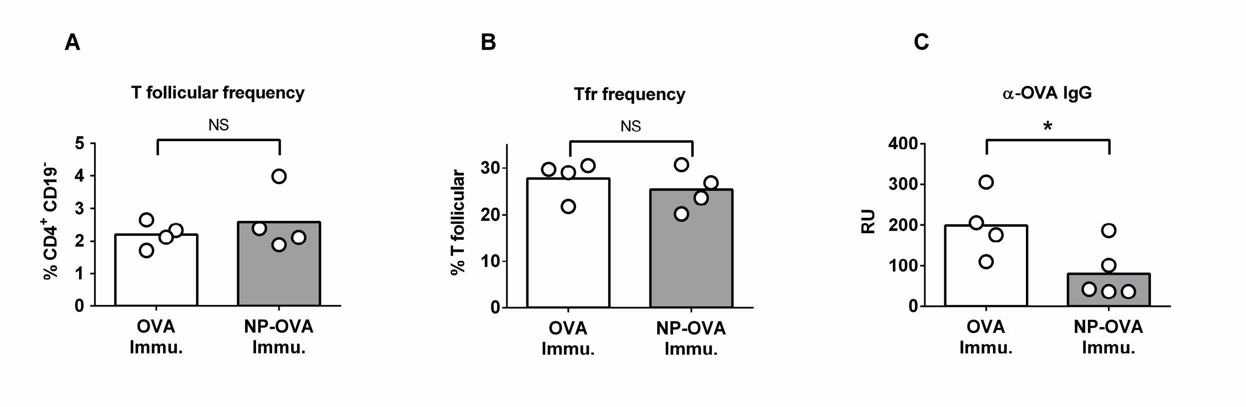

Figure S1. NP-OVA and OVA immunized mice have similar T follicular responses, related to Figure 1.
(A-B) Quantification of T follicular cells (A) and T follicular regulatory cells (B) $CD4^+$, $CD19^-$, $CXCR5^{hi}$, $PD1^{hi}$ and $CD4^+$, $CD19^-$, $CXCR5^{hi}$, $PD1^{hi}$ $Foxp3^+$.
(C) Anti-OVA serum IgG Ab response quantified by ELISA on day 10 after immunization.
Bars represent mean; NS, not significant; *$p < 0.05$ unpaired student's *t* test.

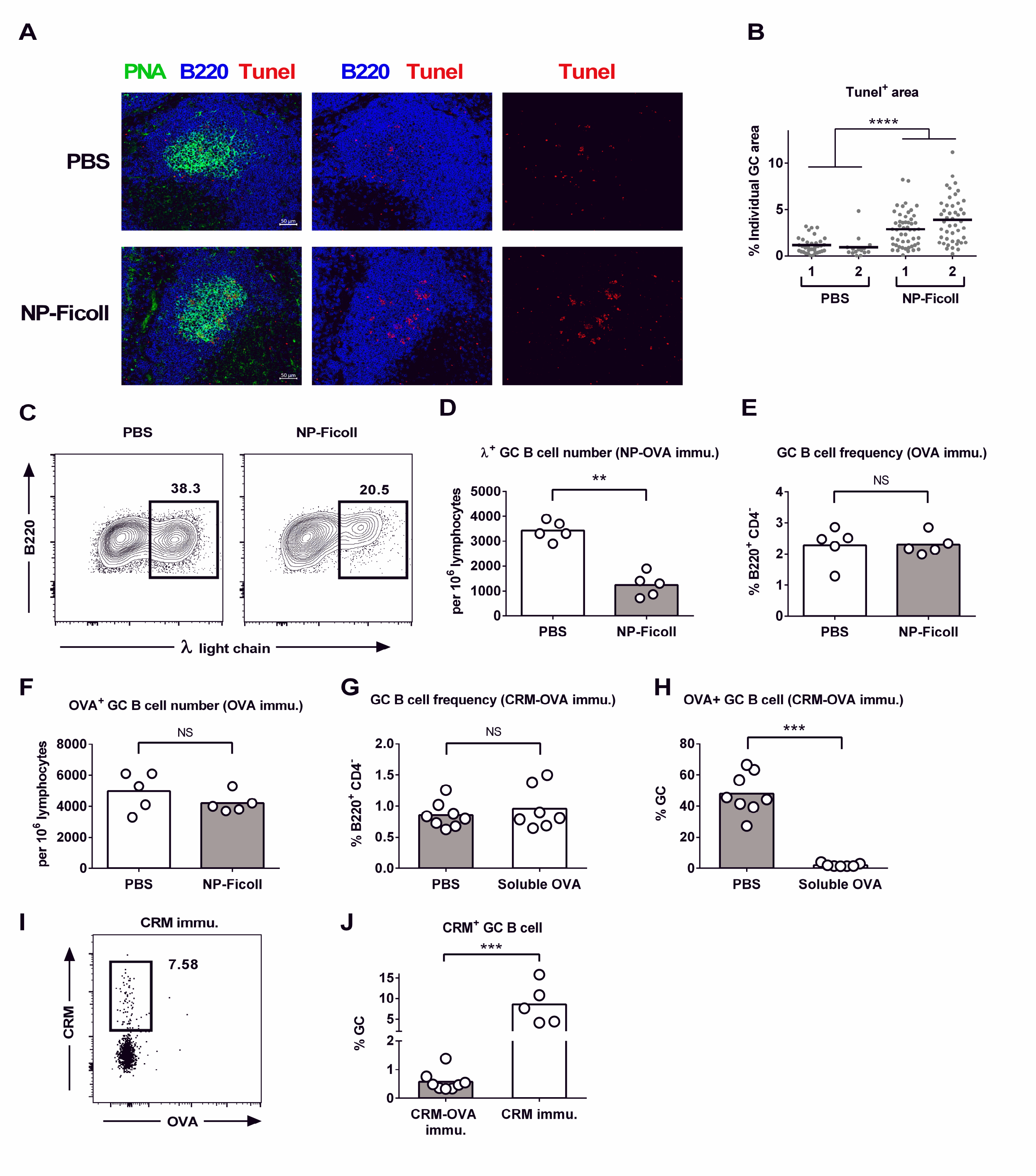

Figure S2. Antigen specific reduction of GC B cells, related to Figure 2.

(A-D) Mice were immunized and treated as in Figure 2A. (A) Representative tunel staining of GCs 6hr after NP Ficoll treatment (B) Quantification of tunel+ area in individual GCs of control on NP-Ficoll treated mice. (C) Representative gates of λ^+ GC B cells ($B220^+$, $GL7^+$, $CD4^-$, $CD38^{lo}$, λ^+) and quantification (D).

(E-F) Mice were immunized with unconjugated OVA in alum and treated with NP-Ficoll as in Figure 2A. Quantification of total GC B cells (E) and OVA-specific GC B cell number (F) after NP-Ficoll treatment (day 9).

(G, H) Mice were immunized i.p. with 1µg of CRM-OVA then treated with soluble OVA or PBS on day7. Spleens were dissected 12 days after immunization. (G) Quantification of total GC frequency and OVA specificity (H)

(I, J) Representative flow plot of mice immunized with unconjugated CRM and quantification of GC specificity (day 12) (J). Bars represent mean; NS, not significant; $^{**}p < 0.01$, $^{***}p < 0.001$, $^{****}p < 0.0001$ unpaired Welch's *t* test.

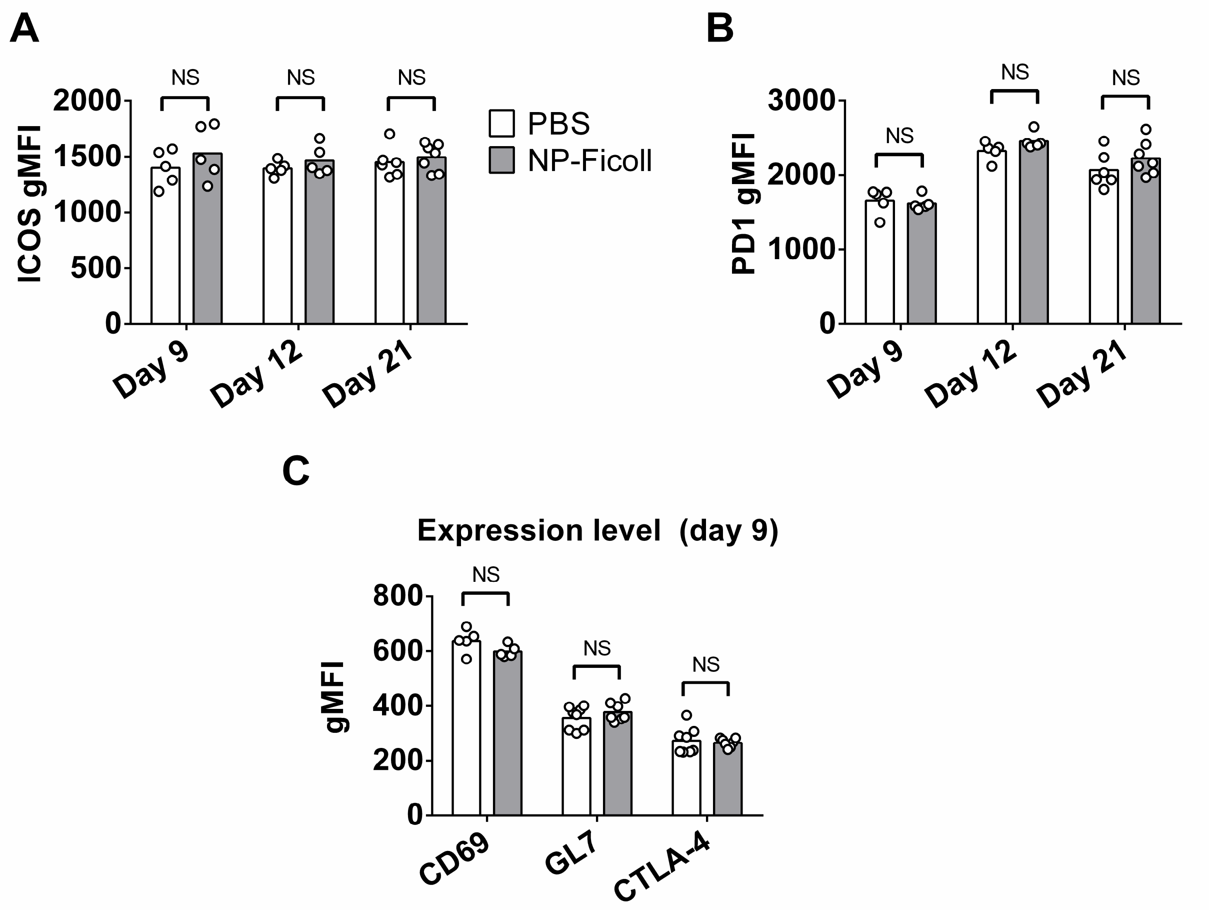

Figure S3. T follicular activation status after NP-Ficoll treatment, related to Figure 3.
(A-C) Mice were immunized and treated as in Figure 2A. T follicular cell $CD4^{+}$, $CD19^{-}$, $CXCR5^{hi}$, $PD1^{hi}$ expression level of various activation markers at indicated time points.
Bars represents mean; NS, not significant unpaired Welch's *t* test.

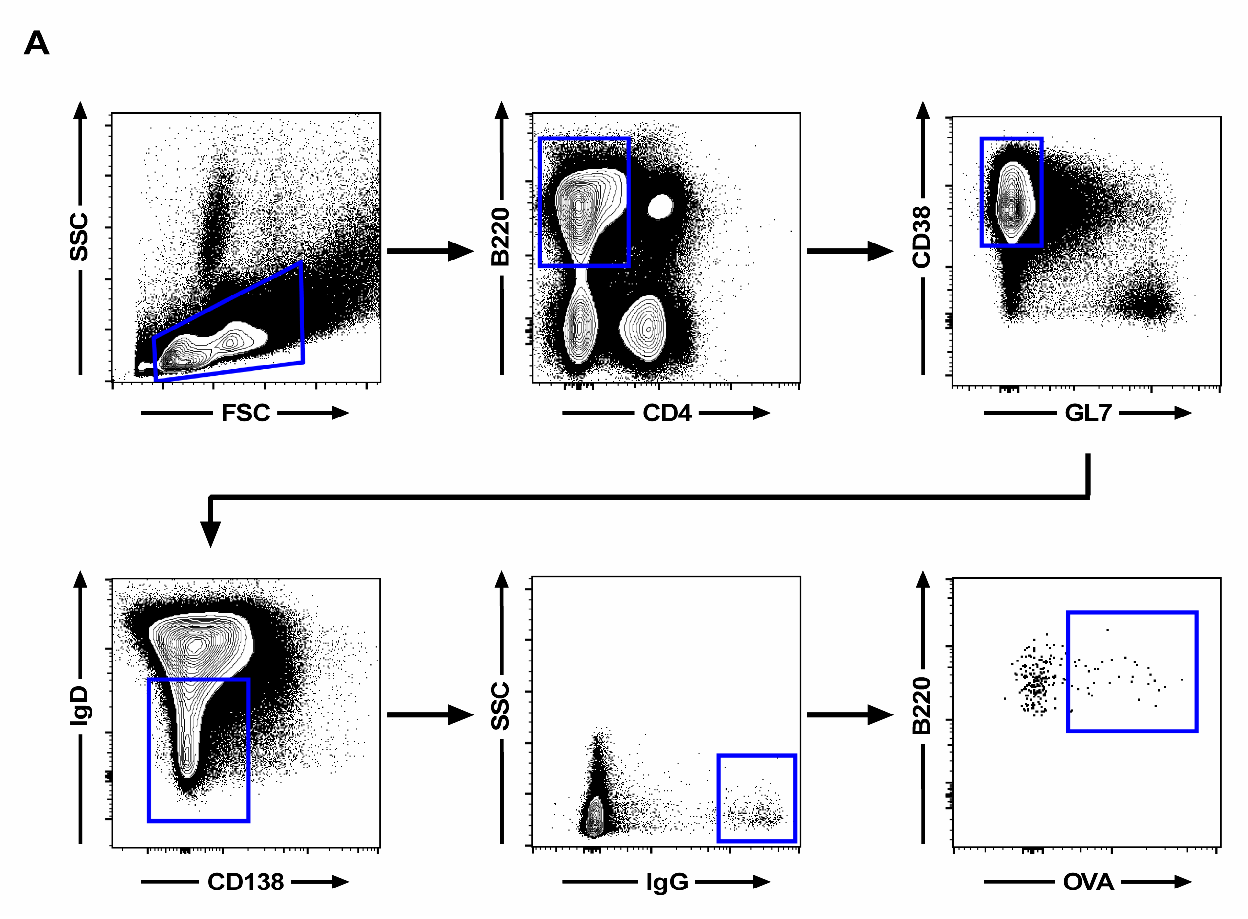

Fig. S4 OVA-specific memory B cell gating, related to Figure 4.
Mice were immunized and treated as in Figure 2A
(A) Gating strategy for OVA+ memory B cells.
